# Lord Lister: XI. London's Indifference to Listerism

**Published:** 1918-04-06

**Authors:** 


### LORD LISTER.

### XI.—London's Indifference to Listerism.

In 1877, at the age of fifty, when his great reputation was at its zenith, Lister was invited to leave the scene of his many triumphs and his large and enthusiastic class of students in Edinburgh and to migrate to London, to become Professor of Clinical Surgery at King's College Hospital. The prospect was, of course, alluring. London is not only the financial, but also to a great extent the intellectual, centre of the world. It is the home of the Court, the seat of the Government, the place of assembly of Parliament, and incomparably the greatest city in the world. Moreover, it was the scene of Lister's education, the residence of many of his friends, and, what weighed with him perhaps as much as anything, it was the only important place in the world in which his teaching was not accepted and the antiseptic system was not followed. London is always jealous of the provinces. London has a profound belief in its own superiority over the rest of the world. Londoners believe in their hearts that if a new idea is to illuminate the human race it will take its origin in London, and that any notion that originates in the provinces is not worth attention. London will never believe that it has anything to learn from the provinces, and any improvement that originates in the provinces is received in London with a certain cold scorn until it is adopted by some prominent citizen of London, who is apt to get the credit of not merely adopting it, but of originating it.

London surgeons detested and despised the antiseptic system of Lister. They would not believe that any good thing could come out of Scotland, even though it had been invented there by an Englishman. Owing to the prevailing mode of making appointments to the great London hospitals, it was unusual for a man to arrive at the senior staff until he was sufficiently advanced in life for his receptive days to be past and gone. An old dog cannot learn new tricks. The profession of medicine has always been a very conservative profession. The overwhelming majority of its members are conservative not only in politics, but also in their profession and in their ways of thought; and this is true especially of the elder members of the profession, and more especially of those elder members who have achieved a high position in the profession and attained the senior appointments on the staffs of hospitals. Whatever the reasons, London would have nothing to say to Listerism, and Lister would have been more than human if he had not desired to bring his torch to the one dark spot remaining on the face of the earth, and to be himself the evangel to spread the new and enlightened faith among the remaining heathen. It must have cost him many a pang to tear himself away from the scene of his early labours, his experiments, his successes, and his triumphs; from his immense and enthusiastic classes, from his cordial and friendly colleagues, and his adoring students; but the attractions of the greatest metropolis in the world, the great metropolis of the world, were overpowering, and he went. It is difficult to believe that he never regretted the step.

London is so enormous, the accumulation of ability, attracted to it from all parts of the earth, is so great, that no man can be supreme in London, and even great men are less conspicuous and less prominent in London than anywhere else. When the god Thor arrived at the palace of the giants the king of the giants addressed him thus: "Thou mayest be a great man among the gods, but thou art but a small man here," and thus it is with a provincial celebrity when he comes to London. In Edinburgh Lister was supreme. He was one of the sights of the city. Every visitor to Edinburgh wished to see Lister; every visitor of importance was introduced to him. Edinburgians made way for him as he passed along the streets, and pointed him out to their provincial cousins. In Edinburgh Lister's name was a name to conjure with. He came to London, and forthwith was lost in the crowd. It must have been so in any case and at any time, but in 1877 many of the great men of the nineteenth century were at the height of their careers. Gladstone, Disraeli, Harcourt, Bright, Lowe, Darwin, Huxley, Owen, Herbert Spencer, G. H. Lewes, George Eliot, Carlyle, Stubbs, Froude, Freeman, Wilberforce, Liddon, Spurgeon, Jenner, Gull, Andrew Clarke, Wilks, Moxon, Paget, and Hutchinson were at the height of their careers and of their fame; and though the name of Lister will shine with greater lustre than any of these to future generations, and will be remembered long after most of them are forgotten, yet at the time of Lister's arrival in London these names were all household words in the mouths of all, while Lister's name was known to but very few outside his own profession, and even in his profession was not honoured in London. There was no place in the world in which Lister's arrival would have attracted so little notice, and in which he would have received so little honour in 1877 as London, and the change from Edinburgh was very great, and could not but be mortifying to any man. Truly a prophet is not without honour, but in his own country, and among his own kin, and in his own house.

In place of the Edinburgh lecture theatre, crowded with as many enthusiastic students as could find standing-room, Lister found at King's College from a dozen to a score of lounging, uninterested youths, sprinkled here and there on benches otherwise empty. King's College is an avowedly sectarian institution, enveloped in a religious atmosphere, and accordingly the nursing arrangements at King's College Hospital were in the hands of a religious sisterhood whose members

Previous articles appeared Jan. 26, Feb. 2, 9, 16, 23, March 2, 9, 16, 23 and 30, p. 549.

**LORD LISTER**—(*continued*).

were bound by rules and vows, and thought much more of the rigid observance of their rules and vows than of the welfare of the patients. They assumed to themselves all the authority of ecclesiastical arrogance, and instead of the nurses being subordinate to the medical staff, the medical staff found themselves subordinate to the nurses. In place of his half-a-dozen wards with their sixty or seventy patients at Edinburgh he had at King's College Hospital but two wards with a maximum capacity of two dozen patients, and these wards were of course empty when he first arrived. The whole surgical profession of London was utterly indifferent where it was not actively antagonistic to Listerism. Students found that familiarity with the antiseptic system told against them in their examinations by men who despised and detested it, and Lister found himself *vox clamantis in deserto.*

In strange contrast with the apathy and indifference, if not the active antagonism, that he met with in London was the reception accorded to him on a visit about this time to the Continent. In 1879 he attended the International Medical Congress, which met in that year in Amsterdam, and the following account is taken from the pages of the by no means friendly *British Medical Journal.*

"Professor Lister was received by the whole Congress with an enthusiasm that knew no bounds. When he stepped forwards to the desk to open his address, the whole assembly rose to their feet, and, with deafening and repeated rounds of cheers, waving of hats and handkerchiefs, hailed the distinguished professor of King's College with acclamations, renewed minute after minute and time after time, as his name was again shouted forth by some grateful and enthusiastic acolyte. This remarkable scene—unprecedented, we imagine, in the history of medical science—continued for some minutes, until Professor Donders, the President, advancing with the distinctive grace and dignity for which he is remarkable, and taking Professor Lister by the hand, as he stood overwhelmed by this magnificent ovation, obtained a moment's silence, and, addressing him, said: 'Professor Lister, it is not only our admiration we offer you, it is our gratitude, and that of the nations to which we belong.'" Others besides Lister, who, as a Quaker, was very familiar with the text of Scripture, must have uttered to themselves the familiar passage already quoted, that a prophet is not without honour, but in his own country, and among his own kin, and in his own house.

A word may be said here in addition to what has been said in a previous paper, of the difference between antisepsis and asepsis. Antisepsis is the destruction of pathogenic microbes in and about a wound. Asepsis is the prevention of such microbes from gaining access to a wound. It is obvious that the one is the complement of the other, and that both are founded on the basic principle that suppuration and other ill results in a wound are due to the invasion of pathogenic microbes. Not only are the two systems, if they can be called two systems, not antagonistic, but each is the necessary complement of the other. The two names have, however, been applied to two methods of carrying out in practice the same principle. The name antisepsis is applied to the use of chemical microbicides which shall destroy or inhibit the microbes that might otherwise gain access to the wound: the name asepsis is applied to the use of dressings that are sterile, but have been sterilised by heat, and do not contain any antiseptic or bactericide. That is the whole difference between what are pompously called two different systems. The difference in principle is little or nothing. The difference in practice is considerable, for it is manifest that dressings, including protective towels, swabs, and so forth, that are merely sterilised and contain no germicide are incessantly liable to accidental contamination, and when contaminated may be fatal in use; while dressings that are charged with a germicide are almost free from the danger of contamination, since they contain within themselves that which counteracts and destroys any slight or moderate contamination to which they may be subjected. The practical difference is that the aseptic system, thus conducted, requires incessant and extreme vigilance, care, and precaution in order to prevent contamination: the antiseptic system frees the surgeon from much of this pre-occupation, and leaves him at liberty to devote almost the whole of his attention to the operation itself. At the same time it reduces to a much lower minimum the risks to the patient. The aseptic system, thus understood, is really a legacy from the opponents of Listerism, who ignorantly believed that if they could obtain Lister's results without the use of carbolic acid they were triumphantly refuting Lister. Of course, they did no such thing. They merely confirmed his principle, while substituting for the detail of his practice another detail much more troublesome and more dangerous.

(*To be continued.*)

